# The Effect of (−)-Epigallocatechin-3-Gallate Non-Covalent Interaction with the Glycosylated Protein on the Emulsion Property

**DOI:** 10.3390/polym11101688

**Published:** 2019-10-15

**Authors:** Haiying Feng, Hua Jin, Yu Gao, Xiuqing Zhu, Qingshan Zhao, Chunhong Liu, Jing Xu

**Affiliations:** 1 College of Science, Northeast Agricultural University, Harbin 150030, China; 18745724605@163.com (H.F.); jinhua@neau.edu.cn (H.J.); gaoyu1914775129@163.com (Y.G.); liuchunhong@neau.edu.cn (C.L.); 2 College of Food Engineering, Harbin University of Commerce, Key Laboratory of Grain Food and Comprehensive Processing of Grain Resource, Harbin 150076, China; 3 Laboratory Management Office, Northeast Agricultural University, Harbin 150030, China; zhaoqingshan@neau.edu.cn

**Keywords:** black bean protein isolate, (−)-epigallocatechin-3-gallate, glycosylation, non-covalent interaction, emulsion properties

## Abstract

The effect of (−)-epigallocatechin-3-gallate (EGCG) on protein structure and emulsion properties of glycosylated black bean protein isolate (BBPI-G) were studied and compared to native black bean protein isolate (BBPI). The binding affinity of BBPI and BBPI-G with EGCG belonged to non-covalent interaction, which was determined by fluorescence quenching. EGCG attachment caused more disordered protein conformation, leading to a higher emulsification property. Among the different EGCG concentrations (0.10, 0.25, 0.50 mg/mL), the result revealed that the highest level of the emulsification property was obtained with 0.25 mg/mL EGCG. Therefore, the BBPI-EGCG and BBPI-G-EGCG prepared by 0.25 mg/mL EGCG were selected to fabricate oil-in-water (O/W) emulsions. After the addition of EGCG, the mean particle size of emulsions decreased with the increasing absolute value of zeta-potential, and more compact interfacial film was formed due to the higher percentage of interfacial protein adsorption (AP%). Meanwhile, EGCG also significantly reduced the lipid oxidation of emulsions.

## 1. Introduction

The native protein has been widely used as an emulsifier to prepare O/W emulsions on account of good surface activity [1]. Moreover, in order to enhance the physical stability and oxidation stability of emulsion, modification of native proteins was often carried out to inhibit the flocculation, fat floating and lipid oxidation [2]. As reported, the protein–polysaccharide complex can effectively promote the protein to adsorb to the oil–water interface more firmly through the hydrophilic groups of polysaccharides and improve the emulsion stability [3]. Furthermore, Yang et al. [4] obtained the conjugates via the Maillard reaction between soybean protein isolate and the soybean-soluble polysaccharide, which showed higher physical stability of O/W emulsion-containing citrate.

At the same time, the high surface area of O/W emulsions makes it easy to oxidize in contact with oxygen for the lipid. Although protein can form a protective film around the oil droplets to delay the oxidation of oil, their limited antioxidant capacity is not entirely satisfactory. In general, phenols are considered to suppress the oxidation and protect the interfacial structure of emulsions. EGCG has strong radical scavenging ability and antioxidant activity due to the polyhydroxy characteristic, and it has been widely used in the research and development of functional food currently. For instance, Fan et al. [5] found that the combination of whey protein and EGCG effectively improved oxidation stability of herring oil emulsion. Liu et al. [6] reported that the porcine bone protein hydrolysate (PBPH)-rutin conjugate could increase the emulsion stability and oxidation stability, which might be ascribed to the formation of a dense network structure at the oil–water interface. In addition, the combination of glycosylation and phenolic modification could react synergistically or additively to increase the emulsions stability [7]. Liu et al. [8] studied the application of a ternary conjugate of chlorogenic acid (CA), lactoferrin (LF) and dextran (Dex) as emulsifiers in β-carotene emulsions under different processing and storage conditions. The study showed that the ternary conjugates showed better emulsifying properties and antioxidant activities than the native protein alone or the CA-LF and LF-Dex conjugates, which also improved the physical and chemical stability of β-carotene emulsions. This was because proteins were used to offer surface activity, polysaccharides offer strong spatial repulsion, and polyphenols provide antioxidant activity.

In food systems, polyphenols and proteins can be combined by non-covalent action and covalent action. The non-covalent binding of plant polyphenols and proteins is interacted through hydrogen bond, hydrophobic interaction, π bond, ionic bond, and other non-covalent bonds [9,10]. The non-covalent binding of protein to polyphenols causes changes in protein structure, which can enhance the functional properties of proteins. Jiang et al. [11] found that the non-covalent interaction between chlorogenic acid and whey protein (WPI) or casein (CS) resulted in the partial unfolded structure of the protein, so that the solubility and foaming ability of WPI and CS developed. Karefyllakis et al. [12] demonstrated that the non-covalent interaction between sunflower protein isolate (SFPI) and chlorogenic acid had a positive effect on the interface and emulsification properties of SFPI. Moreover, since polyphenols could not be oxidized in the non-covalent interaction, the non-covalent complex formed by proteins and polyphenols had definite antioxidant capacity. Staszewski et al. [13] reported that the initial droplet size and stability of the emulsion prepared with the β-lactoglobulin-green tea polyphenols complex at pH 6 were better than β-lactoglobulin, and the presence of polyphenols also improved the oxidation stability of liver fish oil.

Many studies have demonstrated the effect of phenolic compounds on the structure and properties of proteins. However, few studies have addressed the effect of structural changes caused by the non-covalent interaction between phenolic compounds and glycosylated proteins on the properties of emulsions. Therefore, in order to improve the emulsifying and antioxidant properties of proteins, this paper discussed the effect of EGCG addition on the structure and emulsifying property of BBPI grafted to glucose. BBPI-EGCG and BBPI-G-EGCG non-covalent complexes were characterized by fluorescence quenching analysis, FTIR and emulsibility (EAI, ESI). Then, the properties of emulsions were analyzed using the mean particle size, zeta-potential, AP%, TEM, and oxidative stability (POV and TBARS).

## 2. Materials and Methods

### 2.1. Materials

BBPI were obtained from Hei Long Jiang Agriculture Company Limited (Hei Long Jiang, China). Glucose was obtained from Baolingbao Biology Company (Shandong, China). (−)-Epigallocatechin-3-gallate (EGCG) was bought from Yuanye Biotechnology Co. LTD (Shanghai, China). Soya oil of first grade gold arowana was purchased from Yihai Kerry foodstuffs marketing company (Jiangsu, China). All other chemicals were of analytical grade.

### 2.2. Preparation and Characterization of Maillard Reaction Products

The BBPI was glycosylated with glucose in the ratio of 2:1 (BBPI:G, w/w) at 80 °C for 4 h in the phosphate buffer (0.1 M, pH 7.0) and then freeze-dried. The Maillard reaction products were named as BBPI-G. In our previous study [14], the graft degree (DG) of glycosylated black bean protein under this condition was 18.83%. The BBPI-G was characterized by SDS-PAGE and fluorescence spectroscopy.

### 2.3. Incubation of BBPI and BBPI-G with EGCG

BBPI and BBPI-G were dispersed in phosphate buffer solution (0.01 M, pH 7.0), respectively. EGCG stock solution was prepared in phosphate buffer (0.01 M, pH 7.0). The protein solution and glycosylated protein solution (4 mg/mL) were intensively mixed with EGCG (0.10, 0.25, and 0.50 mg/mL), respectively, and incubated at room temperature (25 °C) for 2 h to obtain BBPI-EGCG and BBPI-G-EGCG non-covalent complexes [15]. Finally, prepared solutions were freeze-dried for later use.

### 2.4. Fluorescence Spectroscopy

The EGCG solution with different concentrations was added into the protein solution and mixed thoroughly to ensure the protein concentration in each mixture was 0.2 mg/mL. Then, mixtures were incubated in constant temperature water bath at 25 °C for 5 min. The fluorescence spectra of BBPI-EGCG and BBPI-G-EGCG were recorded using a F-4500 fluorescence spectrophotometer (Hitachi, Tokyo, Japan) at the excitation wavelength of 280 nm, and the emission spectra was 300–550 nm.

In order to study the mechanism of interaction, the fluorescence quenching of BBPI-EGCG and BBPI-G-EGCG was researched. Fluorescence quenching includes dynamic quenching and static quenching. Firstly, the type of fluorescence quenching was determined by the Stern-Volmer equation [16]:

(1) $$F_{0}/F=1+K_{q}\tau_{0}\left[Q\right]=1+K_{sv}\times \left[Q\right]$$

Here, *F*_0_ and *F* are the fluorescence intensities before and after the added quencher (EGCG); *K*_q_ is the fluorescence quenching rate constant. τ_0_ is the average life of fluorophore without a quencher in the biomacromolecule (τ_0_ = 10^−8^ s). [Q] is the concentration of the quencher (EGCG), and *K*_sv_ is the Stern-Volmer quenching constant.

And then, the binding constant *K*_a_ and the number of binding sites (*n*) can be calculated according to a double-logarithmic equation [17] as follows for the static quenching:

(2) $$log\left[\frac{F_{0}-F}{F}\right]=logK_{a}+nlog\left[Q\right]$$

Here, the slope of the double logarithmic plot yields the number of binding sites (*n*) and the intercept provides the binding constant (*K*_a_).

### 2.5. Fourier Transform Infrared Spectroscopy (FTIR)

The infrared spectra of different samples were determined by Bruker Vertex 70 FTIR spectrometer (Bruker Optics, Ettlingen, Baden-Württemberg, Germany). The infrared spectrum with a wave number range of 4000–400 cm^−1^ was determined with a resolution of 4 cm^−1^ and a scanning frequency of 64 times. The curve fitting process of the FTIR spectra was computed by Peakfit Version 4.12 (SeaSolve software company, San Jose, CA, USA) [14].

### 2.6. Emulsifying Properties

The determination of the emulsifying properties was based on the classic turbidimetric method of Pearce and Kinsella [18] with slight modifications: Taking a 12 mL protein solution (1 mg/mL) and adding 4 mL of soybean oil. After being homogenized at 10,000 rpm for 2 min, 40 μL was absorbed from the bottom immediately at 0 min and 10 min, diluted with 5 mL SDS (0.1%, w/w) and shook evenly. Then the absorbance was measured at 500 nm. The calculational formula of emulsification (EAI, ESI) was as follows:

(3) $$\mathrm{EAI}\left(m^{2}/g\right)=\frac{2\times T\times A_{0}\times N\times 10^{-4}}{\varphi \times L\times c}$$

(4) $$\mathrm{ESI}\left(\min\right)=\frac{A_{0}}{A_{0}-A_{10}}\times t$$

where T is 2.303, A_0_ and A_10_ are the absorbance of the emulsion at 0 min and 10 min after homogenization, N is the dilution factor (125), φ is the volume of the oil phase (0.25), L is the path length of cuvette (1 cm), and c is the concentration of each protein sample before being homogenized.

### 2.7. Emulsion Preparation

According to the method of Jin et al. [19], O/W emulsions were prepared containing aqueous phase (2% emulsifier, w/v) and 3% oil phase (soybean oil, v/v) by ultrasonic method with appropriate modifications. Firstly, different prepared samples (BBPI, BBPI-EGCG, BBPI-G, and BBPI-G-EGCG) were dissolved in phosphate buffer (0.01 M, pH 7.0) to dissolve them entirely. The aqueous phase and the oil phase (soybean oil) were pre-mixed (magnetic stirring) at room temperature for 10 min. And then, the crude emulsion was prepared by using a homogeniser (FJ200-SH, Shanghai specimen model factory, Shanghai, China) at 10,000 rpm for 4 min. Final emulsion was obtained by using an ultrasonic processor (Ningbo Xinzhi Biotechnology Co. Ltd., Ningbo, China) under ultrasonic power of 400 W and ultrasonic time of 15 min.

### 2.8. Mean Particle Size and Zeta Potential of Emulsions

Emulsions were diluted 100 times with phosphate buffer (0.01 M, pH 7.0) to ensure sample uniformity and avoid multiple scattering effects. The mean particle size and polydispersity index (PDI) of emulsions were measured by dynamic light scattering instrument (Malvern Nano-S90, Malvern Instruments, Worcestershire, UK). The zeta-potential of emulsion was measured by particle microelectrophoresis (Zetasizer Nano Z, Malvern Instruments, Worcestershire, UK).

### 2.9. Determination of the Percentage of the Interfacial Protein Adsorption (AP%)

According to the method of Chen et al. [20], AP% of various emulsions was determined with appropriate modifications. The emulsion was centrifuged at 10,000 g for 60 min, which showed the two phases: the cream layer at the top and the aqueous phase at the bottom, and then a disposable syringe was used to suck the subnatant carefully and filter it through a 0.45 μL filter membrane. The protein concentration of the filtered subnatant (C_f_) was determined by Lowry method [21]. The protein concentration of the initial protein dispersion (C_0_) and the centrifugal supernatant (C_s_) were determined under the same centrifugation conditions. The AP% was as follows:

(5) $$\mathrm{AP}\%=\frac{C_{s}-C_{f}}{C_{0}}\times 100\%$$

### 2.10. Transmission Electron Microscope (TEM) Observation

The microstructure of emulsions on the oil–water interface was studied by TEM. A drop of diluted emulsions was dropped into a copper mesh of 200 meshes and adsorbed for 15 min. And then the phosphotungstic acid solution (2%, v/v) was stained on the copper mesh for 10 min. After the copper mesh was dried, the TEM images were recorded by H-7650 electron microscope (JEOL, Tokyo, Japan) under the acceleration voltage of 100 kV.

### 2.11. Oxidative Stability

The oxidation stability of emulsions was evaluated by peroxide value (POV) of the initial oxidation product and thiobarbituric acid reactive substances (TBARS) of the secondary oxidation product (0, 2, 4, 6, 8, and 10 days).

The method of POV was from Chaiyasit et al. [22] with some modifications: Taking 0.2 mL emulsion and adding it into a 1.5 mL mixture solution of the isooctane and isopropanol (3:1, v/v). The mixed solution was swirled for 1 min and centrifuged at 5000 g for 30 min. And then, the 0.2 mL supernatant was taken to mix with 2.8 mL mixture solution of the methanol and n-butyl alcohol (2:1, v/v), and the mixture of 15 μL ammonium thiocyanate of 3.94 M and 15 μL ferrous solution was added to react. The absorbance was measured at 510 nm after reacting for 20 min under the room temperature (25 °C). Hydrogen peroxide concentration was obtained using the standard curve of cumene hydroperoxides.

According to the method of Mei et al. [23], a 2 mL emulsion was mixed with 2 mL thiobarbital acid solution. Heated mixture solutions were placed in a boiling water bath for 15 min and then cooled to the room temperature. After centrifugating at 1000 g for 10 min, the absorbance of the supernatant was measured at 532 nm. The standard curve of 1, 1, 3, 3-tetraethoxypropane (malondialdehyde; MDA) was used to determine TBARS.

### 2.12. Statistical Analysis

Three batches of samples were prepared, and all measurements were made in triplicate. The results were expressed as average value ± standard deviation. SPSS 20.0 software was used for evaluation and analysis. The significant differences between the average values were analyzed by one-way analysis of variance (ANOVA) (*p* < 0.05) and independent sample T test (T) (*p* < 0.05).

## 3. Results and Discussion

### 3.1. Characterization of Maillard reaction Products

**Figure 1:**
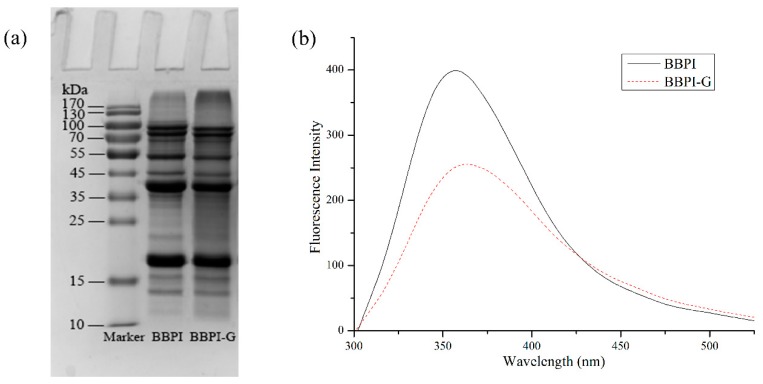
Characterization of BBPI-G. SDS-PAGE (**a**) and Fluorescence spectra (**b**) of BBPI and BBPI-G.

BBPI-G covalent complex was prepared by Maillard reaction with wet heat method. As shown in Figure 1a, BBPI was grafted with glucose-forming BBPI-G conjugates, which could be confirmed by the high molecular weight bands which appeared on the top of the stacking gel. This result indicated that the amino group of BBPI formed a covalent bond with the carbonyl group of glucose and a new covalent complex was formed by Maillard reaction [24].

As shown in Figure 1b, the fluorescence intensity of BBPI-G covalent complex produced by Maillard reaction decreased more significantly than native BBPI, which might be due to the interaction between the hydroxyl group in glucose and tryptophan residues in black bean protein, resulting in a certain shielding effect. It accorded with the fluorescence characteristics of Maillard reaction products. At the same time, the emission wavelength of BBPI-G was bathochromic shifts. This phenomenon suggested that the tryptophan hydrophobic chromophores inside the protein molecules were more exposed to the protein surface. Then, the surrounding environment of tryptophan residues became more polar, so that the red shift of fluorescence emission wavelength occurred after Maillard reaction [25].

### 3.2. Fluorescence Quenching Analysis

**Figure 2:**
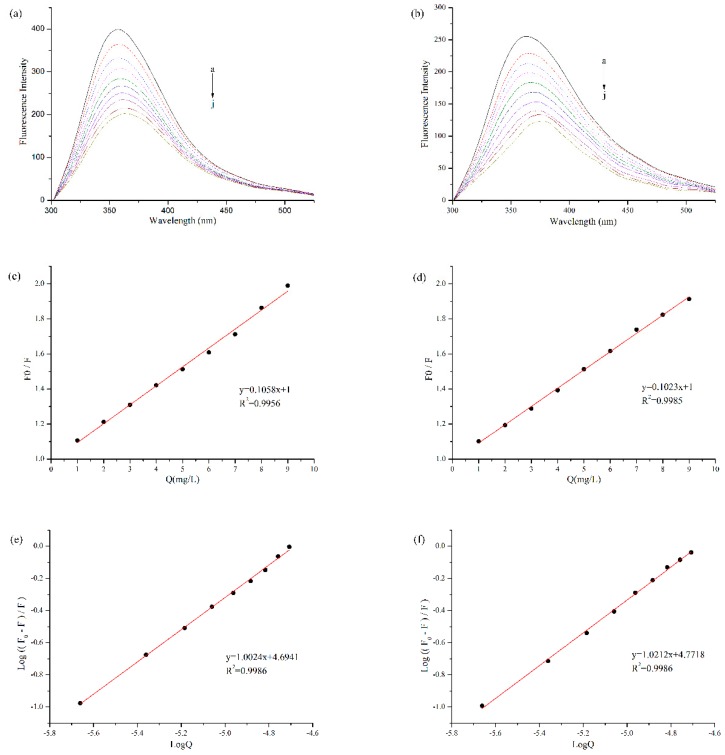
Fluorescence quenching analysis of BBPI (**a**) and BBPI-G (**b**) treated with different concentrations of EGCG (a–j: 0, 1, 2, 3, 4, 5, 6, 7, 8, and 9 mg/L) at pH 7.0. The Stern-Volmer plots to determine K_sv_ of BBPI (**c**) and BBPI-G (**d**). The Double logarithm plot to determine K_a_ and n of BBPI (**e**) and BBPI-G (**f**).

Fluorescence quenching was often used to measure the binding affinity between protein and polyphenol. Protein is a fluorophor, and its endogenous fluorescence mainly comes from amino acid residues such as Tryptophan (Try), Tyrosine (Tyr), Phenylalanine (Phe) and so on [11]. Among them, Try and Tyr residues are excited to produce fluorescence at the wavelength of 280 nm [26]. As shown in Figure 2a,b, with the addition of EGCG, the fluorescence intensity of BBPI and BBPI-G both declined significantly. It was ascribed to the lower quantum yields induced by hydrophilic polyphenol, which led to the fluorescence quenching of tryptophan via the polyphenol attachment to the protein molecules [16]. On the other side, the fluorescence spectra of BBPI and BBPI-G showed a slight red shift after the addition of EGCG, which implied that the proteins were unfolded. This result caused the amino acid residues to be exposed to the protein surfaces, facing a more polar environment.

According to the Stern-Volmer equation, with [Q] as the independent variable and F_0_/F as the dependent variable, Figure 2c,d were obtained by linear fitting. The quenching constants (K_sv_) (the slope of the straight line) were, respectively, 4.85 × 10^4^ M^−1^ and 4.69 × 10^4^ M^−1^ for BBPI-EGCG and BBPI-G-EGCG. The homologous K_q_ values of BBPI-EGCG and BBPI-G-EGCG were 4.85 × 10^12^ M^−1^·S^−1^ and 4.69 × 10^12^ M^−1^·S^−1^. In general, the maximum dynamic quenching rate constant K_q_ of all kinds of quenching agents for biological macromolecules was 2.00 × 10^10^ M^−1^·S^−1^. If the quenching constant became lager than this value, it could be considered as static quenching [27]. Herein, these K_q_ values were much larger than the maximum dynamic quenching constant, which indicated that the BBPI or BBPI-G fluorescence quenching caused by EGCG belonged to the static fluorescence quenching. This revealed that the adsorptions in this work should be caused by forces such as hydrophobic interactions between molecules, instead of the dynamic fluorescence quenching induced by diffusion and collision of molecules [28]. Hence, from the results of fluorescence spectrum, it could be known that the binding of BBPI or BBPI-G with EGCG was a non-covalent interaction in our experiment conditions.

Since static quenching was the main reason for fluorescence quenching of BBPI-EGCG and BBPI-G-EGCG, the binding constant (K_a_) and its number of binding sites (n) could be calculated by using the double logarithmic equation. As shown in Figure 2e,f, K_a_ of BBPI-EGCG was 4.94 × 10^4^ M^−1^ and n was 1.00. Then, K_a_ of BBPI-G-EGCG was 5.91 × 10^4^ M^−1^ and n was 1.02. This result showed that K_a_ (both in the 10^4^ range) and n of BBPI-G-EGCG were similar with those of BBPI-EGCG, which indicated that BBPI grafted with glucose did not affect the binding of protein and EGCG [17].

### 3.3. Secondary Structure Changes of BBPI-EGCG/BBPI-G-EGCG

FTIR spectroscopy was generally used to monitor proteins’ secondary structure changes. As provided in Table 1, compared with BBPI, the ordered structure (α-helix structure + β-sheet structure) content of BBPI-G decreased significantly, while the disordered structure (β-turn structure + random coil structure) content increased significantly. The results showed that the secondary structure of BBPI changed significantly after Maillard reaction and turned to a more disordered direction [14]. This may be due to the fact that the interaction between BBPI and polysaccharide molecules affected the hydrogen bonding and van der Waals forces between molecules, which broke up the stability of the protein secondary structure [29]. Therefore, the secondary structure of protein molecules became more disordered. Based on this, the content of ordered structure continually decreased, and the content of disordered structure continually increased after the addition of EGCG, while the changes became even more violent with the increase of EGCG concentration. This indicated that the protein molecules underwent the unfolding and rearrangement via the EGCG combination and transformed into the looser and flexible complexes. This is because the protein secondary structure was affected by the hydrophilic and hydrophobic interactions and the hydrogen bond between EGCG and protein molecules, which is consistent with the previous research between EGCG and milk proteins [30].

In the secondary structure of the protein, the α-helix structure is composed of multiple peptide bond planes rotating through the α-carbon atoms and tightly coiled into a stable right-handed helix. Meanwhile, all peptide bonds in the peptide chain can form hydrogen bonds, so the α-helix is a very stable secondary structure [31]. The β-sheet structure relies on hydrogen bonds formed between C=O and H in two peptide chains or within one peptide chain to maintain conformational stability. Although all the peptide bonds are involved in the formation of interchain hydrogen bonds in the β-sheet structure, they are not as stable as the α-helix structure [32]. From Table 1, in the ordered structure, the content of α-helix decreased and that of β-sheet increased with the binding of EGCG, which implied that EGCG induced the continuous transformation of the α-helix structure to β-sheet [16]. This result suggested that binding with EGCG further induced a looser protein structure due to the stretching of amide I (C=O of the peptide bond) in the ordered structure [33]. Therefore, it manifested that glucose and EGCG had a certain synergistic effect on promoting more disordered protein. Similar results were reported by Jia et al. [16] that chlorogenic acid, ferulic acid and epigallocatechin-3-gallic acid ester altered the secondary structure of β-lactoglobulin and induced the conversion of α-helix into β-structure. Shen et al. [28] studied the non-covalent interaction between tea polyphenols and two typical egg albumin proteins and they also found that the β-sheet structure of both proteins increased, and the α-helix structure decreased, which resulted in a more loosely structured protein.

### 3.4. Emulsifying Properties of BBPI-EGCG/BBPI-G-EGCG

**Figure 3:**
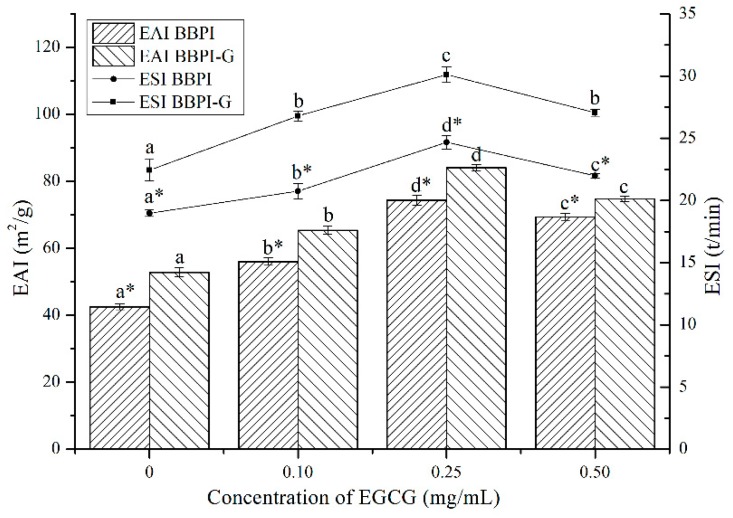
Emulsifying properties (EAI and ESI) of the BBPI and BBPI-G with different concentrations of EGCG prepared oil-in-water emulsions. a–d means with different letters differ significantly in the same protein with different concentrations of EGCG (*p* < 0.05). * means differ significantly between BBPI and BBPI-G (*p* < 0.05).

BBPI are considered as amphipathic molecules containing hydrophilic and hydrophobic groups, in oil–water system, hydrophilic groups with the water molecules and hydrophobic groups with oil molecules. As shown in Figure 3, after the Maillard reaction, the EAI and ESI of BBPI increased from 42.53 m^2^/g and 18.98 min to 52.82 m^2^/g and 22.44 min, respectively. This may be because glucose was a hydrophilic sugar with a polyhydroxy structure and the –OH group of glucose in BBPI-G promoted the adsorption between proteins and water molecules through its hydrogen bonding ability [14]. At the same time, based on the infrared data above, we could obtain that the covalent grafting of the sugar chain led the protein structure to be more disordered, which optimized the emulsification performance [4].

As shown in Figure 3, after EGCG addition, EAI of BBPI-EGCG and BBPI-G-EGCG increased to 74.39 m^2^/g and 84.14 m^2^/g and ESI increased to 24.67 min and 30.12 min, respectively. Since the binding with EGCG enhanced the surface hydrophilic and hydrophobic of the protein, its interfacial activity was enhanced and the adsorption speed of oil–water interfacial increased, which was conducive to forming a tight and thick emulsion base [34]. However, excessive concentration of EGCG combined with BBPI or BBPI-G reduced EAI and ESI slightly, although still being higher than that of BBPI or BBPI-G. This could be attributed to the high concentration of EGCG affecting the thickness of the interface film and causing slight coalescence and flocculation of emulsions [35]. Li et al. [35] studied the effects of zein hydrolysate and sage extract on emulsification and oxidation stability of myofibrillar protein-stabilized O/W emulsions, which had a similar result that the emulsifying property of the protein showed a trend of increasing first and then decreasing with the increase of SE concentration. The above results showed that the glycosylation and non-covalent binding to EGCG both resulted in higher EAI and ESI. Hence, it could be found that the two methods could improve the protein emulsibility synergistically.

### 3.5. Mean Particle Size and Zeta-Potential of Emulsions

According to the determination results of EAI and ESI, BBPI and BBPI-G had the best emulsification properties with the EGCG concentration of 0.25 mg/mL. Therefore, this condition was selected to prepare emulsions. The determination results of mean particle size and zeta-potential of emulsions in Table 2 could more intuitively reflect the effect of EGCG addition on the physical stability of the emulsion system. In general, the oil droplets easily maintained a homogeneous distribution with small particle size. Therefore, evaluating the mean particle size is one of the main methods to analyze the stability of different emulsion samples. The mean particle size of freshly prepared emulsions stabilized by BBPI, BBPI--EGCG, BBPI-G, and BBPI-G-EGCG were 291.30 ± 0.56, 267.47 ± 0.38, 282.73 ± 0.35, and 245.59 ± 0.71 nm, respectively. The mean particle size of emulsions with BBPI-G, BBPI-EGCG and BBPI-G-EGCG were lower than that with BBPI. And the emulsion with BBPI-G-EGCG had the smallest mean particle size, which was consistent with the emulsification activity. Among them, BBPI-G with polysaccharide attachment could form a macromolecular stable layer around oil droplets and maintain stability through spatial repulsion [4]. On the other hand, according to the above discussion, the structure of BBPI became more disordered due to glycosylation, which also improved the emulsification performance of proteins. As a result, the average particle size of emulsions decreased, thus furnishing a better emulsion stability. After, the non-covalent reactions with EGCG, BBPI-EGCG and BBPI-G-EGCG were more easily adsorbed on the oil–water interface, which may be due to the increased amphiphilicity and the decreased interfacial tension of proteins [6].

The zeta-potential mainly describes the properties of the electrostatic potential around the surface of particles [7]. The zeta potential of freshly prepared emulsions stabilized by BBPI, BBPI-EGCG, BBPI-G, and BBPI-G-EGCG were −24.47 ± 0.23, −28.30 ± 0.11, −23.20 ± 0.17, and −26.30 ± 0.26 mV, respectively. Compared with BBPI, the emulsion with BBPI-G had a slight decrease in absolute values of zeta-potential, which might be due to the apparent charge shielding effect of the addition of glucose chains [36]. On the contrary, the binding of the protein with EGCG led to an increase in the electric potential. This suggested that the corresponding electrostatic repulsion force was increased, which led to greater separation distances between droplets, and this was conducive to form more stable oil droplets in emulsions, thus increasing the physical stability of emulsions with BBPI-EGCG or BBPI-G-EGCG [5]. This result was consistent with the data of mean particle size. Accordingly, it could be realized that the combination of glucose and EGCG both brought positive effects to the physical stability of O/W emulsions.

### 3.6. Interfacial Protein Adsorption Fraction of Emulsions

The properties of emulsions were also affected by AP% and the interactions between the free proteins in the aqueous phase. The percentage of the Interfacial Protein Adsorption (AP%) of the four emulsions is shown in Table 2. As can be observed, there were relatively few proteins on the interface membrane, and AP% of BBPI, BBPI-EGCG, BBPI-G, and BBPI-G-EGCG were 9.52 ± 0.29, 12.41 ± 0.19, 10.53 ± 0.38, and 17.54 ± 0.29, respectively. After glycosylation, the affinity between proteins and hydrophilic polysaccharides in BBPI-G increased through the Maillard reaction covalent bonding [4]. Sequentially, this promoted protein anchoring at the oil–water interface and the formation of thicker film at the interface, causing the increase of AP% in BBPI [37].

AP% in BBPI-EGCG and BBPI-G-EGCG increased continually, because the binding of EGCG was in favor of protein adsorption to the O/W interface. Sabouri et al. [38] discovered that the interaction between EGCG and caseins at the oil–water interface increased the expansion modulus of the EGCG–protein layer at the interface, and sodium caseinate emulsion was used as the carrier of EGCG, so that the complex formed at the interface affected the physical and chemical properties of emulsions. Wang et al. [39] investigated the possibility of constructing an algal oil delivery nanoemulsion system with the colloid complex of zein hydrolysate (ZH) and tannic acid (TA). The emulsions stabilized by ZH-TA complex had better physical stability and higher oxidation resistance. Therefore, the increase of AP% could effectively improve the interfacial thickness of emulsions and facilitate the preparation of the emulsion with better physical stability.

### 3.7. Transmission Electron Microscope Observation of Emulsions

**Figure 4:**
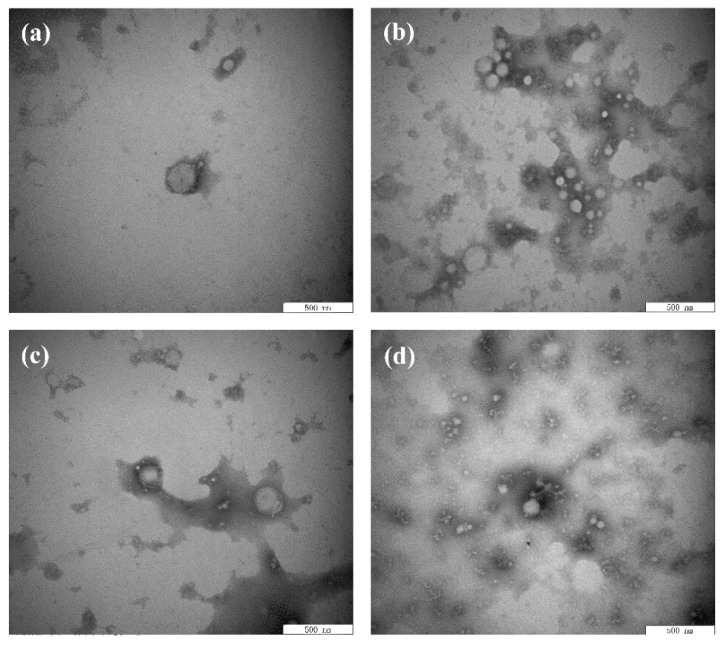
Transmission electron microscope images of the BBPI (**a**), BBPI-EGCG (**b**), BBPI-G (**c**), and BBPI-G-EGCG (**d**) of prepared oil-in-water emulsions.

The microstructure of the emulsion was observed by TEM because the properties of the emulsion were affected by the thickness and compactness of the interface structure. As shown in Figure 4a, the oil droplets were surrounded by BBPI to form an interface film, which was the microstructure of a typical O/W emulsion. Meanwhile, the accumulation of oil droplets was effectively prevented via electrostatic repulsion and spatial steric resistance induced by emulsifier layers [40]. As shown in Figure 4b–d, the microstructure of emulsions made of BBPI-EGCG, BBPI-G and BBPI-G-EGCG all showed a tighter network structure. As glucose was covalently attached to BBPI, the protein structure was stretched to form a space structure to increase the protein adsorption, thus making up the compact membranes on the oil–water interface [4].

Moreover, the interfacial membrane of the emulsion made of BBPI-G-EGCG had the closest network structure, and the results were consistent with mean particle size, zeta-potential and AP%. This could be attributed to the fact that polyphenolic molecules were able to change the protein structure of proteins into a more irregular and disordered state, which led to the frequent adsorption and tight package of protein–polyphenol compounds on the protein surface [39]. In addition, the tight network structure could be considered as a physical barrier to inhibit the dispersion of oxidants in the emulsion, thus preventing the oxidization of emulsions. Zhang et al. [41] researched the effect of the combination of phenolic compounds (licorice extract, LE) with pea protein or pea hydrolyzed protein on the properties of O/W emulsions. The results demonstrated that LE adsorbed on the oil droplets directly or by binding with the protein, thus producing a thick and compact interfacial film and improving the antioxidant properties of emulsions. Jiang and Xiong [42] suggested that the protein had greater interfacial activity and superior emulsion stability when combined with polysaccharides or polyphenols, because the addition of phenols and glycosylation could make the protein molecular have a higher molecule weight and increased the steric hindrance of the protein emulsifier layer.

### 3.8. Oxidative Stability of Emulsions

**Figure 5:**
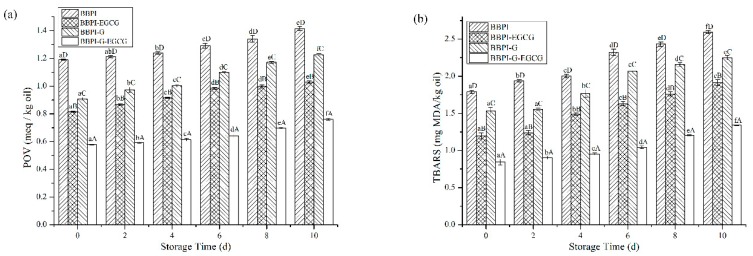
The POV (**a**) and TBARS (**b**) of the BBPI, BBPI-EGCG, BBPI-G, and BBPI-G-EGCG prepared oil-in-water emulsions stored at 25 °C 10 days. a–f means with different letters differ significantly in the same emulsion with different days (*p* < 0.05). A–D means with different letters differ significantly in the same day with different emulsions (*p* < 0.05).

The POV and TBARS are common, sensitive and economical methods for monitoring oil oxidation quality. As shown in Figure 5a,b, the oxidation stability of emulsions prepared by BBPI, BBPI-EGCG, BBPI-G, and BBPI-G-EGCG was evaluated during the 10 days storage at room temperature. From Figure 5a, the POV value of emulsions increased with time extending. After 10 days of storage, the POV value of emulsions prepared by BBPI, BBPI-EGCG, BBPI-G, and BBPI-G-EGCG was 1.41 ± 0.01, 1.03 ± 0.01, 1.23 ± 0.01 and 0.76 ± 0.01 meq/kg oil, respectively. The oxidation degree of BBPI-G emulsion was obviously lower than that of BBPI emulsion. This phenomenon revealed that the emulsion prepared by BBPI-G was more stable against oxidation. This was consistent with the discovery of fish O/W emulsion prepared by glycosylated protein [43]. This result could be attributed to the fact that a dense protective film was formed after glycosylation of the protein, which was effective in isolating oxygen. Compared with emulsions without EGCG, the addition of EGCG significantly reduced the POV value of emulsions as BBPI-EGCG and BBPI-G-EGCG. The latter BBPI-G-EGCG was even lower than the former BBPI-EGCG. EGCG was rich in phenolic groups, which could effectively remove free radicals and chelate transition metals [44]. Then, the oxidative stability of the emulsion system was improved with the BBPI-G-EGCG emulsifier. On the other hand, the protein and polyphenols combined to form a denser interface membrane than BBPI-G and BBPI, which could inhibit the diffusion of oxidants in emulsions more effectively. In brief, it was realized that protein could possess high level capacity to resist the production of hydroperoxides in emulsions via both EGCG conjugation and glycosylation, so that BBPI-G-EGCG has the highest oxidation stability for the emulsion [42].

In Figure 5b, during the 10-day storage period, the TBARS values of different emulsions were BBPI > BBPI-G > BBPI-EGCG > BBPI-G-EGCG, which showed the same trend with POV values. Fan et al. [5] proved that whey protein with EGCG conjugation significantly improved the physical stability of emulsions and inhibited the oxidation of oil, and the POV and TBARS in emulsions system were reduced obviously. In addition, the increase of interfacial protein content could lead to the increase of interfacial membrane thickness, forming a tighter interfacial membrane, and then protecting oil from oxidation [41]. The increase of interfacial membrane protein content also increased the efficiency of glucose and EGCG in inhibiting lipid oxidation on the interfacial membrane.

## 4. Conclusions

In this work, EGCG interacted with BBPI or BBPI-G through non-covalent interaction. Both glycosylation and the addition of phenols tended to make protein structures disordered and loose. After the protein was combined with EGCG, the emulsification activity and emulsion stability of BBPI and BBPI-G were significantly improved. By selecting the best EGCG concentration to make O/W emulsions, we found that the emulsion prepared from BBPI-G-EGCG had the best emulsifying performance due to the synergistic effect of hydrophilic glucose chains and EGCG, which promoted the adsorption capacity of proteins at the interface. Therefore, the emulsion stabilized by BBPI-G-EGCG showed the best stability with the smallest droplet size, higher zeta-potential, highest AP%, and tightest interface membranes. Meanwhile, the emulsion prepared with BBPI-G-EGCG had the best oxidation stability. On the one hand, EGCG had its own oxidation resistance which could inhibit oil oxidation. On the other hand, the increased antioxidation was attributed to an increase in the thickness of interface layers as the AP% increased, which created a tighter network structure to prevent the entry of oxidants. Therefore, Maillard reaction and the addition of EGCG can be used in protein to improve the emulsification and enhance the oxidation stability of products.

## Figures and Tables

**Table 1:** Contents of different secondary structures of BBPI and BBPI-G with different concentrations of EGCG.

		0	0.10 mg/mL	0.25 mg/mL	0.50 mg/mL
**BPPI**	α-Helix (%)	16.84 ± 0.12 ^d^	15.96 ± 0.07 ^c^	15.41 ± 0.14 ^b^	15.10 ± 0.05 ^a^
	β-Sheet (%)	38.29 ± 0.08 ^a^	38.63 ± 0.07 ^b^	39.02 ± 0.04 ^c^	39.17 ± 0.06 ^d^
	β-Turn (%)	28.74 ± 0.10 ^a,^*	29.14 ± 0.10 ^b,^*	29.22 ± 0.07 ^b,^*	29.46 ± 0.08 ^c,^*
	Random coil (%)	16.13 ± 0.04 ^a^	16.26 ± 0.06 ^a,b^	16.35 ± 0.16 ^b^	16.26 ± 0.07 ^a,b^
**BBPI-G**	α-Helix (%)	15.94 ± 0.08 ^c,^*	15.02 ± 0.10 ^b,^*	14.85 ± 0.09 ^b,^*	14.46 ± 0.11 ^a,^*
	β-Sheet (%)	35.99 ± 0.06 ^a,^*	36.60 ± 0.08 ^b,^*	36.51 ± 0.08 ^b,^*	36.80 ± 0.08 ^c,^*
	β-Turn (%)	33.77 ± 0.08 ^a^	33.98 ± 0.07 ^b^	34.04 ± 0.06 ^b^	34.19 ± 0.05 ^c^
	Random coil (%)	14.30 ± 0.10 ^a,^*	14.41 ± 0.10 ^a,b,^*	14.60 ± 0.11 ^c,^*	14.54 ± 0.04 ^b,c,^*

^a–d^ means with different letters differ significantly in the same protein with different concentrations of EGCG (*p* < 0.05). * means differ significantly between BBPI and BBPI-G (*p* < 0.05).

**Table 2:** Characteristics (mean particle size, PDI, zeta-potential and AP) of emulsions stabilized with BBPI, BBPI-EGCG, BBPI-G, and BBPI-G-EGCG.

	Mean Particle Size (nm)	PDI	Zeta-Potential (mV)	AP (%)
**BBPI**	291.30 ± 0.56 ^d^	0.220 ± 0.007 ^c^	−24.47 ± 0.23^b^	9.52 ± 0.29 ^a^
**BBPI-EGCG**	267.47 ± 0.38 ^b^	0.173 ± 0.004 ^a^	−28.30 ± 0.11 ^d^	12.41 ± 0.19 ^c^
**BBPI-G**	282.73 ± 0.35 ^c^	0.187 ± 0.009 ^b^	−23.20 ± 0.17 ^a^	10.53 ± 0.38 ^b^
**BBPI-G-EGCG**	245.59 ± 0.71 ^a^	0.172 ± 0.007 ^a^	−26.30 ± 0.26 ^c^	17.54 ± 0.29 ^d^

^a–d^ means with different letters differ significantly in the same column with different samples (*p* < 0.05).
